# The Aggregate Excess Measure of Severity of Extreme Events

**DOI:** 10.6028/jres.099.054

**Published:** 1994

**Authors:** Clive W. Anderson

**Affiliations:** Department of Probability and Statistics, University of Sheffield, Sheffield, UK

**Keywords:** aggregate excess, extreme event, flood, generalized Pareto distribution, ozone, point process, severity, storm, threshold model, Weibull distribution

## Abstract

It is suggested here that in many environmental and other contexts the severity of an extreme event might usefully be represented by the sum of the excesses of a measured variable over a high threshold. The general form of the limiting distributions of such sums for a wide class of models has been derived by Anderson and Dancy, and has suggested methods for the statistical analysis of data concerning extreme severity. This work is reviewed here, and some extensions to the distributional theory are presented. An application of the methods to atmospheric ozone levels, which calls for the extension of the approach to take account of covariate information is reported.

## 1. Introduction

The severity of a storm or a flood is often a function not only of the peak value of whichever environmental variable is concerned, but also of other aspects of the extreme event, such as its duration and temporal shape. An extended run of days with temperatures just below freezing, for example, can be more disruptive to everyday human activity and to animal and plant life than a single day with a much sharper frost. Similarly, sustained moderately high water levels in a river or the sea can lead to greater flooding than a more extreme level lasting for only a short time. To attempt to analyze such examples in a way which captures the notion of severity implicit in them demands an extension of traditional statistical methods for extremes, which have tended to concentrate largely on the modelling of maxima or storm peaks. In Ref. [1] it was suggested that for an important class of applications a simple way to quantify the idea of severity is in terms of the sum of the excesses of the environmental variable over a high threshold during the extreme event. In the case of a flood, for example, this sum or aggregate excess is a discrete approximation to the total volume of water overtopping the threshold, and in the case of temperatures the analogous quantity defined for low values, the aggregate deficit, is a measure of exposure or cumulative damage. In the earlier paper some distribution theory was developed for aggregate excesses, and an application to flood data was discussed. Here I review that work and present some extensions of its distributional results, and discuss a new application to ozone concentrations.

## 2. Preliminaries

The techniques to be described are related to threshold methods for extremes [2], and the distributional results are formulated in terms of the Mori-Hsing point process representation [3, 4] for the structure of high values of a stationary sequence. We briefly recall ideas from these two areas.

Suppose {*X_j_*} denotes a sequence of observations, and let *u* be a high threshold. Times *j* at which *X_j_ > u* are referred to as *exceedances* of *u* by {*X_j_*}, and the sizes of overshoots *X_j_* − *u* at exceedances are called *excesses* over the level *u*. In environmental applications exceedances are often found to occur in clusters corresponding to physical storms. Threshold methods are based on the modelling of the peak excess within each cluster by a generalized Pareto distribution, with distribution function of the form

$$G\left(x:\zeta \sigma \right)=1-{\left(1+\frac{\zeta x}{\sigma }\right)}^{-1/\zeta },$$ (1)

where *σ* > 0 is a scale parameter, *ζ* (−∞ < *ζ* < ∞) is a shape parameter, and the range of *x* is such that *ζx/σ > −*1.

Let *N* denote the number of exceedances within a cluster, and suppose that ζ_1_ ≥ … ≥ *ζ_N_* are the corresponding excesses. Then the suggestion above is that the aggregate excess within a cluster

$$S=\sum_{1}^{N}\zeta_{j}$$

is for some purposes a reasonable measure of the severity of a storm event. For statistical modelling we are interested in the distribution of *S*, particularly for high thresholds *u*. Since *S > ζ*_1_, we expect *S* to have (in the limit as *u* increases) a tail no lighter than that of the limiting generalized Pareto distribution of *ζ*_1_. The distribution of *S* is also expected to reflect the cluster size and the pattern of dependence between individual excesses *ζ*_1_.

Suppose now that *M_n_* = max_1≤i≤n_ *X_i_*. It is known that for many {*X_i_*} sequences *M_n_* may be normalized to converge in distribution to some nondegenerate limit. Suppose in fact that there is a continuous and strictly decreasing function *u_n_*(*τ*) such that, for each *τ* > 0,

$$\underset{n\to \infty }{\lim}P\left(M_{n}\leq u_{n}\left(\tau \right)\right)=e^{-\tau }.$$ (2)

Let *u_n_*^−1^ denote the inverse function of *u_n_*. Consider now the two-dimensional point process with points $\left(j/n,u_{n}^{-1}\left(X_{j}\right)\right)$. In Ref. [4], which generalizes Ref. [3], it is proved under a weak long-range mixing condition *∆* that if this point process converges as *n*→∞ then its limit has points of the form (*S_i_*, *T_i_Y_ij_*), *i ≥* 1, 1 *≤ j ≤ K_i_*, where (*S_i_*,*T_i_*), *i* ≥ 1 are the points of a unit Poisson process in ℜ^+2^, and for each *i*, {*Y_ij_* : *j* = 1,…, *K_i_*} with *Y_ij_ ≡* 1, is a point process on [1, ∞) with a random number *K_i_* of points. Moreover the processes {*Y_ij_* : *j* = 1,…,*K_i_*) for each *i* are independent of each other and of the {(*S_i_*,*T_i_*)} process, and are identically distributed.

A natural interpretation of this convergence result is that large values of the {*X_i_*} sequence occur in clusters, located in time at the points of a simple Poisson process, and that values within a cluster (from the peak downwards) are given, after transformation, by *T_i_*, *T_i_Y_i_*_2_,… respectively (reading upwards). Note that, since the transformation is decreasing, a cluster *peak* corresponds to the *lower* endpoint of a vertical string of points in the limiting point process.

In what follows it will be convenient to suppose that the point process associated with each cluster contains infinitely many points *Y_ij_* arranged in increasing order of size

$$1\equiv Y_{i1}\leq Y_{i2}\leq \ldots$$

but that infinite values of the *Y_ij_* are allowed after the first point, so that *K_i_*, the number of points in a cluster, is just the index of the last finite *Y_ij_*. By this means stochastic properties of *K_i_* are subsumed no-tationally in those of {*Y_ij_*}.

We are interested in particular in clusters of exceedances by {*X_j_*} of a high threshold *u*. Let $v=u_{n}^{-1}\left(u\right)$. Then *X_j_ >u* is equivalent to $u_{n}^{-1}\left(X_{j}\right)<v$, and so, in the limit, clusters of exceedances of *u* correspond exactly to those clusters in the point process for which *T_i_* < *ν*. Given that we are dealing with such a cluster (as we assume from now on) it follows from the unit Poisson nature of {(*S_i_*, *T_i_*)} that *T_i_* is uniformly distributed over (0, *ν*).

For many {*X_j_*} the transformation *u_n_* is related in a simple way to the marginal distribution function, *F* say, of *X_j_*. Suppose in fact that {*X_j_*}, still satisfying condition ∆, has a positive extremal index 0. Then ([5], Theorem 3.7.2)

$$\underset{n\to \propto }{\lim}P\left(M_{n}\leq u_{n}\left(\tau \right)\right)\underset{n\to \propto }{\lim}F^{n\theta }\left(u_{n}\left(\tau \right)\right).$$ (3)

Hence, if the tail function 1 − *F* of *F* is denoted by *ℱ*, it follows from Eq. (2) that

$$n\theta ℱ\left(u_{n}\left(\tau \right)\right)∼-n\;\theta \;\log\;F\left(u_{n}\left(\tau \right)\right)∼\tau ,$$

for large *n*. We may therefore define *u_n_* by

$$u_{n}\left(\tau \right)={ℱ}^{-1}\left(\tau /n\theta \right).$$ (4)

In particular therefore

nθℱ(u)=v,

and so the excesses within a cluster, in decreasing order of size, are in the limit (dropping the cluster index *i*, no longer relevant)

$$\begin{array}{l} \zeta_{i}=u_{n}\left(TY_{j}\right)-u\\ ={ℱ}^{-1}\left(TY_{j}ℱ\left(u\right)/v\right)-u\\ ={ℱ}^{-1}\left(T^{\prime }Y_{j}ℱ\left(v\right)\right)-u,\\ j=1,2,\ldots ,N \end{array}$$ (5)

where *T′ = T/ν* is uniformly distributed over (0, 1). The aggregate excess for the cluster is

$$S=\sum_{1}^{N}\zeta_{j},$$

where *N*, the number of exceedances in the cluster, is

$$N=\max\left\{j:T^{\prime }Y_{j}<1\right\},$$

and *T′* is independent of the *Y_j_* process.

## 3. Asymptotic Distributions of Aggregate Excess

In this section we outline various asymptotic distributional properties of aggregate excesses which follow from the preceding discussion. The asymptotic distribution of aggregate excess *S* itself turns out to depend on the *Y* process partly through random sums

$$R_{j}=\sum_{i=1}^{j}Z_{ij}$$

where the *Z_ij_*, are defined in terms of {*Y_i_*} by

$$Z_{ij}=[\begin{array}{cc} {\left(\frac{Y_{j+1}}{Y_{i}}\right)}^{\zeta }-1 & \text{for}\;\zeta >0\\ \log\;\left(\frac{Y_{j+1}}{Y_{i}}\right) & \text{for}\;\zeta =0\\ 1-{\left(\frac{Y_{j+1}}{Y_{i}}\right)}^{\zeta } & \text{for}\;\zeta <0. \end{array}$$ (6)

### 3.1 Limit Distributions of *S*

*Suppose that the stationary sequence* {*X_j_*} *satisfies Hsing’s mixing condition ∆ and has positive extremal index, and that the marginal distribution F of the X_j_ is such that the limiting distribution of peak excesses within a cluster is generalized Pareto with shape parameter ζ. Suppose too that the corresponding point process* $\left\{\left(j/n,u_{n}^{-1}\left(X_{j}\right)\right)\right\}$ *converges to a limiting process with the structure described in Sec. 2. Then, as the threshold level u tends to the upper end point, x_+_ say, of the support of X*,

$$\begin{array}{c} \underset{n\to x+}{\lim}P\left(\frac{S}{\gamma_{\zeta }\left(u\right)}>s\right)=\\ {}[\begin{array}{cc} E\left[{\left(\frac{\sum_{1}^{j_{s}}Y_{1}^{-\zeta }}{s+j_{s}}\right)}^{1/\zeta }\right] & for\;\zeta >0\\ E\left[\exp\left(-\frac{s+\sum_{1}^{j_{s}}\log Y_{i}}{j_{s}}\right)\right] & for\;\zeta =0\\ E\left[{\left(\frac{\sum_{1}^{j_{s}}Y_{1}^{-\zeta }}{j_{s}-s}\right)}_{+}^{1/\zeta }\right] & for\;\zeta <0 \end{array} \end{array}$$ (7)

*where*

$$\gamma_{\zeta }\left(u\right)=\{\begin{array}{cc} u & \text{for}\;\zeta >0\\ l\left(1/ℱ\left(u\right)\right) & \text{for}\;\zeta =0\\ x_{+}-u & \text{for}\;\zeta <0 \end{array}$$ (8)

*for a suitable slowly varying function l*, *and*

$$j_{x}=\min\left\{j:Y_{j+1}=\infty \;\mathrm{or}\;R_{j}\geq s\right\}.$$ (9)

*Expectations in Eq. (7) are taken with respect to the point process* {*Y_j_*}.

This result is a consolidation and re-statement of the main limit forms found in Ref. [1]. The proof—essentially a weak convergence argument based on the Mori-Hsing process—exploits regular and slow variation properties of *ℱ*-implied by the assumption that cluster peak excesses are, in the limit, generalized Pareto distributed. For example, when *ζ* = 0, *ℱ* belongs to the domain of attraction of the Gumbel extreme value distribution, so that, as.*x* → ∞,

$${ℱ}^{-1}\left(e^{-w-x}\right)-{ℱ}^{-I}\left(e^{-x}\right)∼w/\left(e^{x}\right)$$

for each *w* > 0, for some slowly-varying function *l* (see, for example, Ref. [6], See. 8,13). Thus

$$\begin{array}{l} \zeta_{j}-1{ℱ}^{-1}\left(T^{\prime }Y_{j}ℱ\left(u\right)\right)-F^{-1}\left(ℱ\left(u\right)\right)\sim \\ \left(-\log\left(T^{\prime }Y_{j}\right)\right)/\left(1/ℱ\left(u\right)\right), \end{array}$$

as *u→x_+_*, which establishes the connection between the limiting behaviour of *S* and the *Y*-process.

We note that Eq. (7) reveals in reasonably explicit form the dependence of the distribution of *S* on the number and pattern of excesses within a cluster.

### 3.2 Joint Limit Distributions

The techniques used to obtain these results may be extended to give limiting distributions for other quantities. As an example (motivated by a question from a reservoir engineer about peak water level and total overtopping discharge at a dam wall) the joint distribution of peak and aggregate excesses is as follows.

*Under the same assumptions as in Sec. 3.1, and with the same notation:*

$$\begin{array}{c} \underset{u\to x+}{\lim}P\left(\frac{S}{\gamma \zeta \left(u\right)}>s,\frac{\zeta_{1}}{\gamma_{\zeta }\left(u\right)}>z\right)=\\ {}[\begin{array}{ll} E\left[\min\left\{{\left(\frac{\sum_{1}^{js}Y_{i}^{-\zeta }}{s+j_{s}}\right)}^{1/\zeta },\left(1+z\right){-}^{1/\zeta }\right\}\right] & for\zeta >0\\ E\left[\min\left\{\exp\left(-\frac{s+\sum_{1}^{j_{s}}\log Y_{i}}{j_{s}}\right),e^{-1}\right\}\right] & for\zeta =0\\ E\left[\min\left\{{\left(\frac{\sum_{1}^{js}Y_{i}^{-\zeta }}{j_{s}-s}\right)}_{+}^{1/\zeta },{\left(1-z\right)}_{+}^{-1/\zeta }\right\}\right] & for\zeta >0 \end{array} \end{array}$$ (10)

Similar joint limiting distributions may also be found for *ζ*_1_ and *S − ζ*_1_. Like Eq. (10) they are singular. Methods of statistical analysis based on them have yet to be explored.

### 3.3 More Explicit Forms for *P*(*S*/*γ_ϵ_*(*u*)*>s*)

**Fig. 1:**
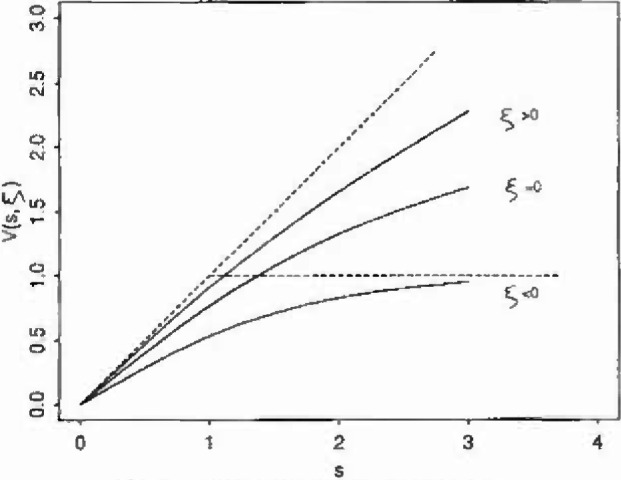
Forms of the *V*(*s*, *ζ*) function.

When specific models are assumed for the *X* process the limiting distributions Eq. (7) take on more explicit forms. Several examples were studied in Ref. [1], Writing

$$\underset{u\to x+}{\lim}P\left(\frac{S}{\gamma_{\zeta }\left(u\right)}>s\right)=\{\begin{array}{ll} {\left(1+\mathrm{sign}\left(\zeta \right)V\left(s,\zeta \right)\right)}^{-1/\zeta } & \text{for}\;\zeta \neq 0\\ \exp(-V\left(s,0\right) & \text{for}\;\zeta =0 \end{array}$$ (11)

it was found that *V*(*s, ζ*) had the same general form in all cases considered: that of a concave increasing function *of s* dominated by *s* when *ζ* > 0, and by min {1, *s*} when *ζ* < 0. See Fig. 1.

The findings and examples above motivate an attempt to fit aggregate excess data by a distribution with tail function of this general form. Two such attempts are described in Sec. 4.

### 3.4 Higher Thresholds

As often in extreme value Statistics, an aim in many applications will be extrapolation to longer time periods or higher levels than seen in data. In particular, for aggregate excesses, extrapolations to higher *thresholds* will often be of interest. For example, in Hood applications knowledge of the aggregate excess above a higher threshold might be vital in estimating the reduction in the size of floods that would result from improved river or sea defences. The following presents a simple relationship on which extrapolation of aggregate excesses could be based.

Suppose that *S_u_* and *S_u′_* denote aggregate excesses above levels *u* and *u*′, respectively, with *u < u′*, in a cluster in which level *u* is exceeded (so that *S_u_*, but not necessarily *S_u_*_′_, is greater than zero). In a slightly more refined notation than used earlier, the limiting forms in Sec. 3.1 are limits, ℋ(*s*) say, of *P*(*S_u_*/*_γϵ_*(*u*) *>s|S_u_* > 0) as *u → x_+_*. We are now interested in *P*(*S_u_/_γϵ_*(*u*) *> s | S_u_* > 0). But

$$\begin{array}{l} P\left(\frac{S_{u^{\prime }}}{\gamma_{\zeta }\left(u\right)}>s|S_{u}>0\right)=\\ P\left(\frac{S_{u^{\prime }}}{\gamma_{\zeta }\left(u^{\prime }\right)}>s\frac{\gamma_{\zeta }(u)}{\gamma_{\zeta }\left(u^{\prime }\right)}|S_{u^{\prime }}>0\right)\\ P\left(S_{u^{\prime }}>0|S_{u}>0\right)\approx \\ ℋ\left(s\frac{\gamma_{\zeta }\left(u\right)}{\gamma_{\zeta }\left(u^{\prime }\right)}\right)P\left(\zeta_{1}>u^{\prime }\right), \end{array}$$ (12)

for high *u*, where *ζ*_1_ is the peak excess in the cluster. Thus the distribution of aggregate excesses with respect to the higher threshold *u′* has a point probability at 0 corresponding to the event *P*(*ζ*_1_ ≤ *u′*) that no exceedance of *u′* occurred, together with a form over the strictly positive half-line which is the the same as that of the original distribution of aggregate excesses except for an increased scale parameter. Estimation of this distribution may therefore be based, through Eq. (12) on estimation of *ℋ* from data on aggregate excesses of *u*, and of *P*(*ζ*_1_ ≤ *u′*) from data on peak excesses of *u* fitted to the Generalized Pareto distribution Eq. (1). Relationship Eq. (12) should also be useful as a means of checking the fit of specific models for *ℋ*, though this aspect has yet to be investigated.

## 4. Applications

### 4.1 Floods on the River Thames

In Ref. [1] an application of some of the limiting results above to data on levels of the River Thames is described. The aim was largely exploratory: to see whether there is support in an important data set for a model of the general kind suggested in Secs. 3.1 and 3,3, and, if there is, to seek an appropriate parametric form for the model. The results were surprisingly positive: confirmation was found for the general form of distribution predicted by asymptotic arguments, and in particular a simple Weibull distribution with

$$P\left(S>s\right)=\exp{\left(-as\right)}^{\phi }),$$ (13)

for some parameters *α* >0 and ϕ was found to give an acceptable fit to data on *S*.

### 4.2 Ozone Concentrations

An analysis of a further set of data, which calls for the extension of the models above to take account of covariate information, is now reported.

The data consist of hourly mean ozone concentrations at a suburban site in Stevenage, about 25 miles north of London, over the years 1978–1989. High levels of ozone are known to cause direct damage to vegetation (see, for example, Ref. [7]). One tentative suggestion is that a plant or tree suffers damage in proportion to cumulative exposure to ozone at concentrations above some threshold. The threshold is not known, and indeed is likely to be different for different plants, but a figure in the range 40 ppb–90 ppb might be plausible. Though this theory is at present no more than a working hypothesis, it prompts an interest in the occurrence of high values of aggregate excesses of ozone concentrations above moderately high thresholds. The analysis summarized below is a preliminary investigation into the possibility of using the aggregate excess models of Sec. 3 to describe such high doses. A more complete account of the biological background, and of the application of the method to spatial variation of exposure over the UK, is given in Ref. [8].

**Fig. 2:**
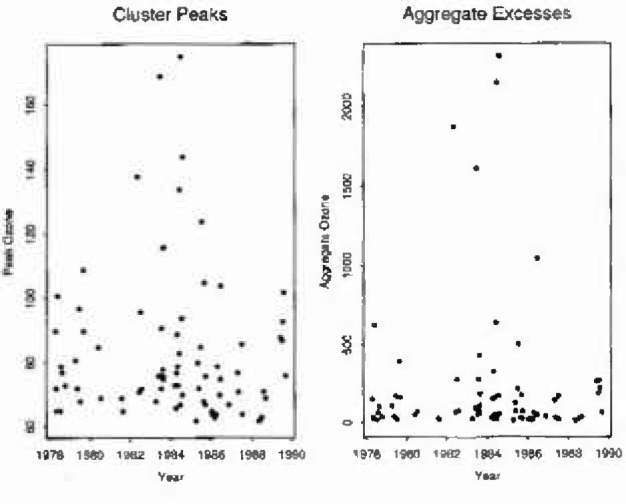
Hourly mean ozone concentrations over 60 ppb: Stcvenage 1978–1989.

For the theory of Secs. 2 and 3 to be applicable it is desirable that we work with independent clusters of high values. The hourly data were therefore subjected to a preliminary declustering procedure, which selected episodes when concentrations above a specified ‘declustering threshold’ were experienced, and ensured that such episodes were separated far enough in lime to give some plausibility to the independence assumption. Figure 2 shows a time plot of the resulting aggregate excesses above a threshold of 60 ppb, obtained with a declustering threshold of 50 ppb and with a time separation between clusters of at least 48 hours—these values being chosen as typical of those of possible scientific interest. An immediate observation from the plot is that the assumption of stationarity is suspect: the middle years 1982–1986 contain some values higher than seen earlier or later. (There are known diurnal patterns in ozone concentrations too, but they are of too short a duration to affect the present analysis.) In view of the apparent nonstationarity a simple model of the kind found useful in the earlier analysis would not on its own be expected to be particularly successful here: and indeed the Weibull model Eq. (13) fitted to aggregate excesses above 60 ppb appears to underestimate the sizes of the highest aggregates.

**Fig. 3:**
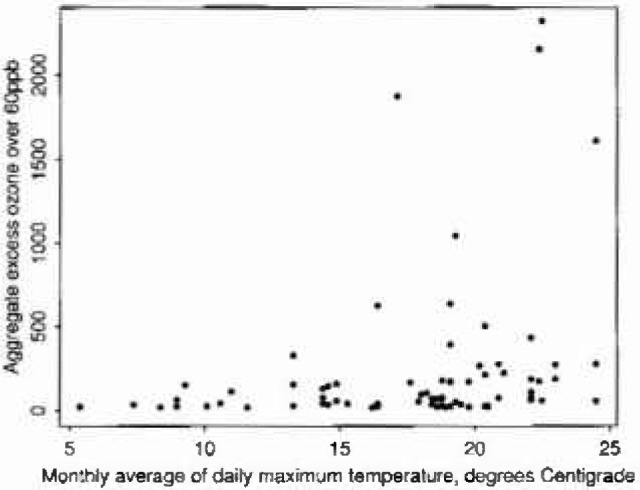
Aggregate excess ozone over 60 ppb vs temperature.

**Fig. 4:**
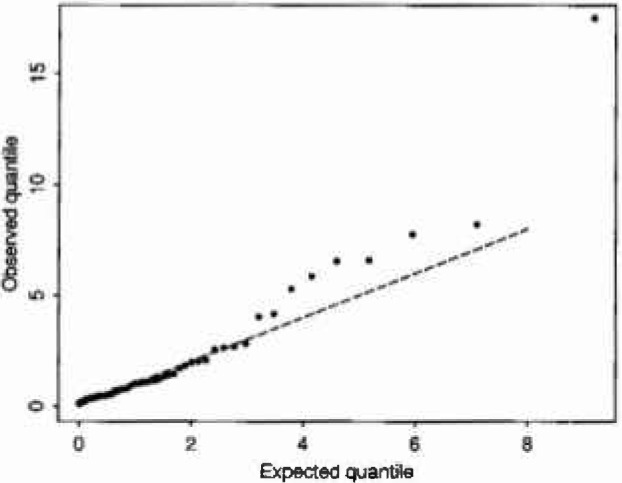
Q-Q plot for aggregate excess ozone: simple covariale model Eq. (14).

**Fig. 5:**
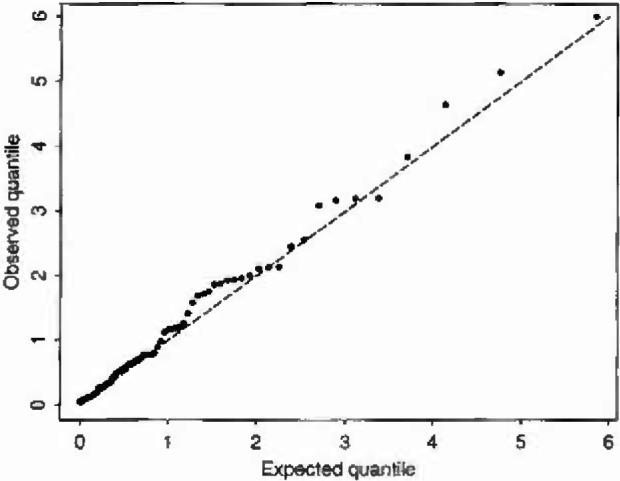
Q-Q plot for aggregate excess ozone: two-type corniate model Eq. (15).

The processes leading to the formation of ozone in the atmosphere are photo-chemical — driven hy strong sunlight. It is possible therefore that unusual weather conditions in the early to mid 1980s may have had some bearing on the possible inhomogeneity. Unfortunately sunlight was not recorded at the Stevenage monitoring site, nor was temperature, which is a crude surrogate for it. Temperature data were not readily obtainable either from nearby meteorological stations, but were to hand for Sheffield, 140 miles north. Figure 3, showing aggregate excesses over 60 ppb against monthly averages of daily maximum Sheffield temperatures, illustrates that in spite of the geographical separation there is nevertheless some connection. It appears that the summers over the relevant years contained some quite warm spells, presumably experienced in Sheffield as well as Stevenage. Accordingly Weibull models which incorporate temperature f as a covariate were fitted. Two forms were used:

$$P\left(S>s\right)=\exp-{\left(s/\delta \left(t\right)\right)}^{\phi },$$ (14)

in which the scale parameter δ depends on *t* in the form *δ*(*t*) = *δ*e*^βt^*; and secondly a model suggested by the evidence from Fig. 3 that not all occurrences of high temperatures *t* at the time of an ozone cluster are necessarily associated with a high aggregate ozone dose. This suggests a model in which ozone clusters are assumed to be of two types, the first showing temperature dependence of the kind above, and the second showing no dependence on temperature. Thus

$$P\left(S>s\right)=\{\begin{array}{cc} \exp-{\left(s/\delta \left(t\right)\right)}^{\phi } & \text{for type}\;1\;\text{clusters}\\ \exp-{\left(s/\delta^{\prime }\right)}^{\phi } & \text{for type}\;2\;\text{clusters} \end{array}$$ (15)

(Since sunlight/high temperature is at best only one of the preconditions known to be necessary for the formation of ozone, there is some general scientific justification for a model of this form.) In fitting, clusters with aggregate excesses above a specified level were taken to be of type 1. A likelihood ratio test shows that model Eq. (15) represents a very worthwhile improvement over Eq. (14) even after allowing for the inclusion of two extra parameters (*W* = 27.26, *p* < 10^−4^, cut-off level for type 1 = 500). Q-Q type plots for the two covariate models are shown in Figs. 4 and 5 respectively. (These are constructed as follows: under model Eq. (14) *S/*(*δ*e*^βt^*) reduces to a standard Weibull variable with unit scale parameter and shape parameter ϕ:*P*(S/(*δ*e*^βt^*)>*s*) = exp(*−s*^ϕ^). Thus a plot of the ordered values of *S/*(*δ*e*^βt^*) from a sample of size *n* against [*−*log(*i*/*n* +1)]^1/ϕ^ should yield an approximate line of unit slope. Figure 4 is a plot of this kind, and Fig. 5 is constructed similarly from model Eq. (15).) Both plots appear to show a quite good fit to the Weibull model after allowing for dependence on temperature, model Eq. (15) doing a little better than model Eq. (14). Further refinements of the models allowing temperature-dependence also of the shape parameter *ϕ* gave no worthwhile improvement in fit as judged by a likelihood test.

Though this is only a preliminary analysis (which we hope to complete with better temperature data), the results so far are encouraging. They appear to show again that models of the form suggested in Sec. 3.3, and in particular a Weibull model —after allowance in this case for nonstationarity—can represent aggregate excess data reasonably well. If this is confirmed, then for example these models will be useful in estimating return levels of future high doses of ozone above 60 ppb or, following the results of Sec. 3.4, above higher thresholds.

## References

[1] Anderson CW, Dancy GP (1992). The severity of extreme events Research Report 92/593 Department of Probability and Statistics.

[2] Davison AC, Smith RL (1990). Models for exceedances over high thresholds (with discussion). J Roy Statist Soc B.

[3] Mori T (1977). Limit distributions of two-dimensional point processes generated by strong-mixing sequences. Yokohama Math J.

[4] Hsing T (1987). On the characterization of certain point processes. Stoch Processes Appl.

[5] Leadbetter MR, Lindgren G, Rootzén H (1983). Extremes and Related Properties of Random Sequences and Processes.

[6] Bingham NH, Goldic CM, Teugcls JL (1987). Regular Variation.

[7] UK Photochemical Oxidants Review Group (1987). Ozone in the United Kingdom.

[8] Smith RI, Anderson CW, Fowler D (1994). Critical levels of ozone over the United Kingdom: mapping aggregate exceedances over moderate to high levels. J Res Natl Inst Stand Technol.

